# A validation of newly developed weight estimating tape for Korean pediatric patients

**DOI:** 10.1371/journal.pone.0271109

**Published:** 2022-07-07

**Authors:** Sungwoo Choi, Sangun Nah, Sumin Kim, Eun O. Seong, So Hyun Kim, Sangsoo Han

**Affiliations:** Department of Emergency Medicine, Soonchunhyang University Bucheon Hospital, Bucheon, Republic of Korea; The University of Mississippi Medical Center, UNITED STATES

## Abstract

Pediatric drug dosages are based on body weight, so accurate measurement thereof is essential. However, this is not possible in emergencies. When using weight-estimating tapes, World Health Organization (WHO) growth charts serve as reference weights; however, local growth charts might be more accurate. This study developed a tape based on 2017 Korean National Growth Charts, which are more suitable for the Korean population than WHO growth charts, and evaluated its performance in a Korean pediatric population. This prospective cross-sectional study analyzed 792 pediatric patients who had visited the emergency department from June 2021 to December 2021. Weights were estimated using the Broselow tape (BT), Pediatric Advanced Weight Prediction in the Emergency Room XL (PAWPER XL), and Body Habitus-based Pediatric Emergency Tape (BHPET). The performance and measurement agreement of the tapes were analyzed. Among the tapes, the BHPET had the smallest mean absolute percentage error (MAPE), of 10.23%, and a root mean square percentage error (RMSPE) of 14.14%. Also, the percentage of weight estimations within 10% of the actual weight (PW10) was 59.6%, indicating better accuracy than the BT and PAWPER XL in all age groups. The intraclass correlation coefficients of the BT, PAWPER XL, and BHPET were 0.931, 0.977, and 0.985, respectively (p < 0.001). The performance and accuracy of the BHPET was similar or slightly superior to that of the other tapes. The body weight estimated using the BHPET for a local pediatric population was sufficiently correlated with the actual body weight.

## Introduction

According to a previous study that analyzed life-threatening medication errors in pediatric patients, the mortality rate was about 10% [1]. Dosing error was the most common cause of medication error, and a life-threatening case was reported up to 24% [2]. Especially, dosing error of up to 65% has been reported even in situations where the correct dosing is critical, such as under resuscitation [3,4]. Dosing error is observed three times more common in pediatrics than in adults, and weight-based dosing system in pediatric patients contributes much to these errors [5,6]. Indeed, weight-related errors have been investigated as one of the most common causes of dosing errors in pediatrics, accounting for more than 30% [7–9]. Also, pediatric medical devices and equipment, and the defibrillation energy dose used for resuscitation, are based on the patient’s weight [10]. As such, the management of pediatric patients is often weight-based, and accurate measurement and documentation of weight is an important issue particularly for pediatric patients [8]. To determine the weight of pediatric patients, direct weighing on an electronic scale device is the best method. However, there is insufficient time to measure body weight when medical intervention or resuscitation is urgently needed. Moreover, depending on the patient’s underlying disease and posture, it may not be possible to use an electronic scale [11,12].

Methods of estimating weight from body length have been developed and are widely used in emergency situations. The Broselow tape (BT) is the gold standard to determine the dose of drugs and appropriate equipment size in emergencies [13]. This tape costs about $25 but it cannot be used if the patient’s height exceeds the length of the tape [14]. Several studies have shown that weight may be under- or overestimated in some populations. An inaccurate drug dose can potentially harm patients [15–18].

The Pediatric Advanced Weight Prediction in the Emergency Room (PAWPER) tape can determine weight more accurately by assessing the patient’s body habitus [19,20]. The PAWPER tape uses World Health Organization (WHO) weight-for-length growth charts as reference weights [19]. However, this tape cannot be used in children taller than about 153 cm. Therefore, the PAWPER XL tape was developed to measure lengths of up to 180 cm and assess body habitus at the 97th and 99th percentiles for length [18].

While the PAWPER XL tape also uses WHO growth charts, there is controversy about its application to certain populations [21]; errors may occur depending on the nationality, ethnicity, and population [22]. Some studies recommend using local growth charts [23–25]. Therefore, this study developed a length-based tape for assessing body habitus using the 2017 Korean National Growth Charts (KNGC), which are more suitable as reference weights for the Korean population than WHO growth charts. We called this tape the Body Habitus-based Pediatric Emergency Tape (BHPET), and evaluated the performance of the BT, PAWPER XL, and BHPET in a Korean pediatric population.

## Materials and methods

### Participants and data collection

This prospective cross-sectional study examined pediatric patients who visited the emergency department of a tertiary university hospital in Gyeonggi-do, South Korea from June 2021 to December 2021. The study enrolled pediatric patients aged from 1 month to 12 years who did not require immediate emergency treatment or intervention. Children shorter than 46 cm or longer than 150 cm, who and thus could not be measured with the BT, were excluded [19], as were those who did not consent to participate in the study or whose height and weight were affected by underlying disease (congenital malformation, neuromuscular disease, genetic disease, etc.).

### Development of the BHPET

**Fig 1:**
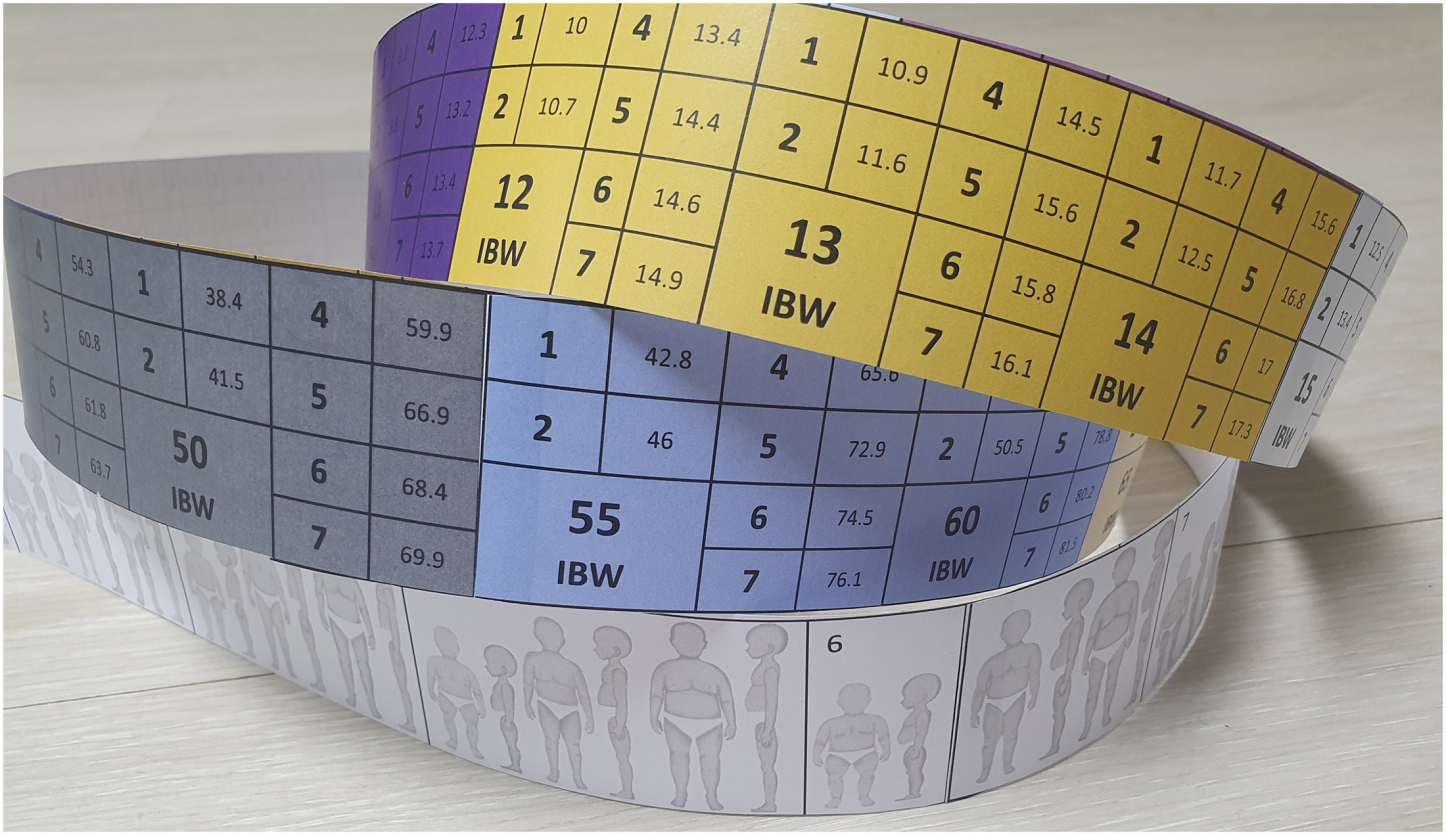
BHPET tape.

The BHPET was developed for assessing body habitus in the South Korean population. Other length-based tapes use WHO growth charts as reference values, which is controversial because of regional variation [21]. The BHPET uses the 2017 KNGC, which is the growth chart published in 2017 by the Korea Center for Disease Control and Prevention (KCDC), as a reference. The KNGC is the growth chart for Korean pediatric population developed by the committee consisting of specialists in pediatrics, family medicine, preventive medicine, statistics, and nutrition and has been released for every 10 years from 1967. The latest version was released in 2017 [26]. Body habitus is scored as follows by an existing system: habitus score (HS) of 1, underweight (5th percentile); HS score of 2, thin (25th percentile); HS score of 3, normal weight (50th percentile); HS score of 4, overweight (75th percentile); and HS score of 5, fat [27]. We added the following: HS score of. 6 (97th percentile), obese; and HS score of 7 (99th percentile), extremely obese. The tape was also designed to measure heights up to 180 cm, to overcome the length limitations of other tapes (Fig 1).

### Measurement of weight

The actual weight and height of the participants were measured when they visited the emergency department by a nurse blinded to the study objectives. Height was measured to within 0.1 cm, and weight to within 0.1 kg. The latter was measured in a standing position (or in a supine position when standing was impossible) using an electronic scale and recorded in the electronic medical records.

The tape measurements were performed in the order BT, PAWPER XL, and BHPET. The same protocol was performed for each tape, by two pediatricians working in the emergency department and blinded to the study objectives. They had received 30 minutes of training on the use of each tape and body habitus scoring before data collection. To minimize bias, they were blinded to the actual weight and height until after the measurements. In the supine position, the starting point for each tape was the top of the participant’s head, and the weight was estimated at the point where the tape passed the heel.

### Data and statistical analysis

Data are expressed as the median with interquartile range (IQR) for continuous variables and as absolute numbers or relative frequencies for categorical variables. The normality of continuous variables was confirmed using the Shapiro–Wilk test. Patients were classified into three subgroups according to age (1–12 months, 2–5 years, and 6–12 years) [28].

The performances of the BT, PAWPER XL, and BHPET were assessed. The mean percentage error (MPE) was the difference between the actual and predicted weights, and was taken to reflect the measurement bias. The mean absolute percentage error (MAPE) and root mean square percentage error (RMSPE) were calculated to assess overall measurement precision. In addition, the percentage of weight estimations within 10% of the actual weight (PW10), and the percentages within 20% (PW20), were calculated as indices of the overall accuracy of the measurements. The intraclass correlation coefficient (ICC) was calculated to compare the actual and estimated weight for each tape; the values were then plotted on a scatterplot. ICC values of < 0.7, 0.7–0.89, and ≥ 0.9 indicated inadequate, good, and excellent agreement, respectively [29]. All statistical analyses were performed using SPSS software (ver. 26.0; IBM Corp., Armonk, NY, USA.). P-values <0.05 were considered statistically significant.

### Ethics approval

The study protocol was approved by our Institutional Review Board (IRB No. 2021-07-005) and conducted in accordance with the Declaration of Helsinki. The purpose of the study was explained to the patients and their parents or legal guardians. Informed consent was obtained from all participants and their parents or legal guardians.

## Results

**Fig 2:**
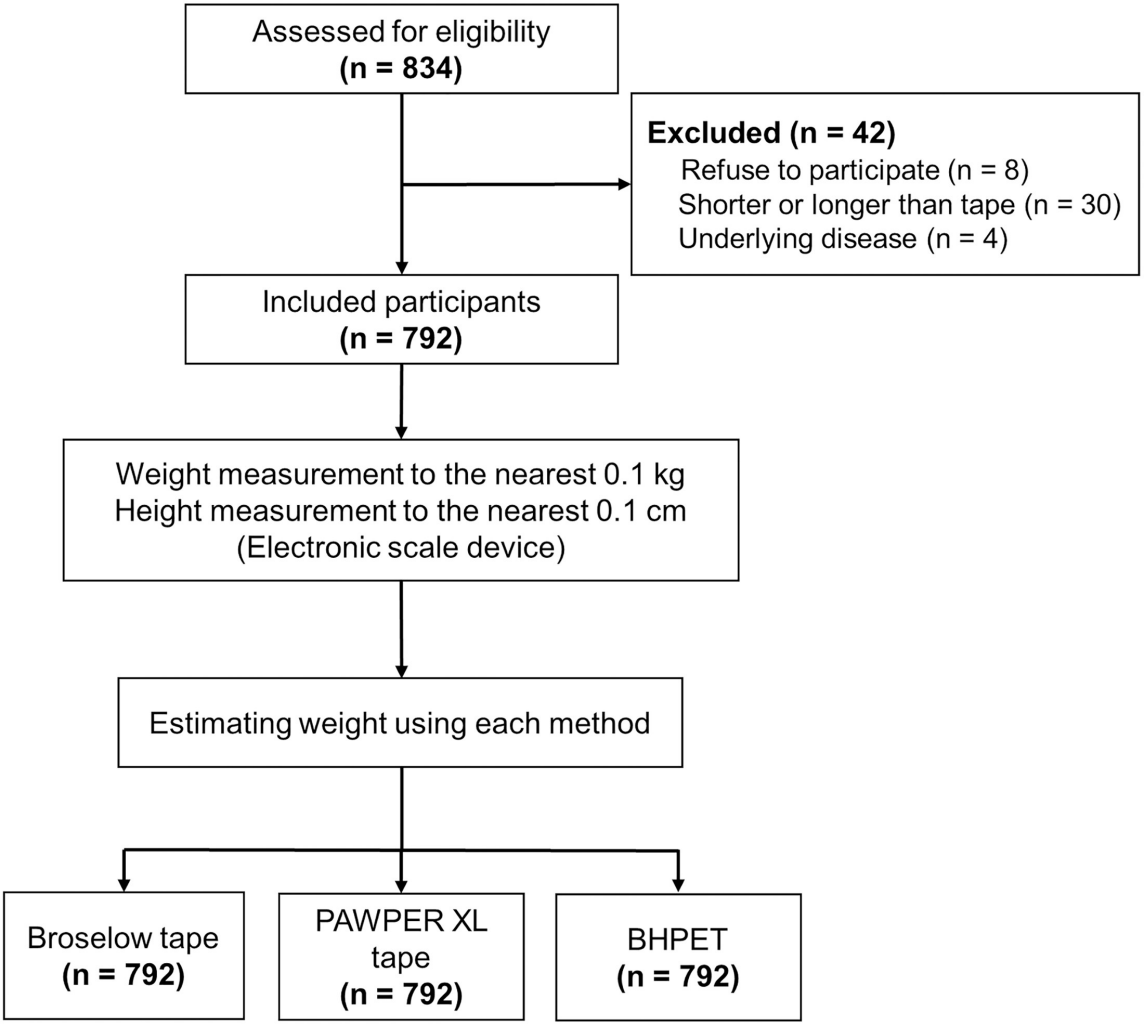
Study population.

The eligibility for participation of 834 pediatric patients who visited the emergency department during the study period was evaluated. Of the patients, 8 who did not agree to participate, 30 who were shorter or longer than the measuring tape, and 4 who had an abnormal body composition or contractures due to an underlying disease were excluded. Ultimately, 792 patients were enrolled (Fig 2).

### General characteristics of the participants

The study enrolled 792 participants [467 (58.3%) males and 334 (41.69%) females]; 82 aged 1–12 months, 449 aged 2–5 years, and 261 aged 6–12 years]. Their average age was 4.19 (IQR: 3.97–4.4) years, the average height was 103.84 (IQR: 102.19–105.5) cm, the average weight was 19.79 (IQR: 18.99–0.6) kg, and the average BMI was 17.13 (IQR: 16.89–17.36) kg/m2. The HS was 2–6 in 83 (10.48%), 524 (66.16%), 140 (17.68%), 37 (4.67%), and 8 (1.01%) patients, respectively (Table 1).

**Table 1:** General characteristics of the participants.

	Total (n = 792)	1–12 months (n = 82)	2–5 years (n = 449)	6–12 years (n = 261)
Age, y	4.19 [3.97, 4.4]	0.52 [0.46, 0.58]	2.62 [2.49, 2.75]	8.04 [7.83, 8.25]
Sex, n (%)				
Male	467 (58.3)	46 (56.1)	260 (57.02)	161 (61.22)
Female	334 (41.69)	36 (43.9)	196 (42.98)	102 (38.78)
Height (cm)	103.84 [102.19, 105.5]	68 [66.07, 69.94]	94.92 [93.74, 96.11]	130.45 [128.94, 131.96]
Weight (kg)	19.79 [18.99, 20.6]	8.28 [7.79, 8.77]	14.81 [14.36, 15.25]	31.99 [30.62, 33.37]
BMI (kg/m^2^)	17.13 [16.89, 17.36]	17.88 [17.1, 18.66]	16.31 [16.05, 16.57]	18.29 [17.84, 18.75]
HS, n (%)				
HS 1	0 (0)	0 (0)	0 (0)	0 (0)
HS 2	83 (10.48)	3 (3.66)	50 (11.14)	30 (11.49)
HS 3	524 (66.16)	48 (58.54)	329 (73.27)	147 (56.32)
HS 4	140 (17.68)	27 (32.93)	62(13.81)	51 (19.54)
HS 5	37 (4.67)	4 (4.88)	8 (1.78)	25 (9.58)
HS 6	8 (1.01)	0 (0)	0 (0)	8 (3.07)
HS 7	0 (0)	0 (0)	0 (0)	0 (0)

Values are expressed as median [interquartile range] or number (proportion).

BMI, body mass index; HS, habitus score.

### Performance of the weight-estimating tapes

The BT had an MPE of –0.41%, MAPE of 12.47%, and RMSPE of 17.19%. The PAWPER XL had an MPE of –1.4%, MAPE of 10.5%, and RMSPE of 14.43%. The BHPET had an MPE of 1.4%, MAPE of 10.23%, and RMSPE of 14.14%. The limits of agreement were –11 to 9.1, –7.11 to 5.55, and –5.41 to 5.66 kg for the BT, PAWPER XL and BHPET, respectively. The PW10 was 51.13%, 59.59%, and 59.6% respectively; the PAWPER XL and BHPET were more accurate than the BT. When analyzed by subgroup, the BHPET had the lowest RMSPE in the 1–12 months group (18.14%) and 2–5 years group (14.28%). In the 6–12 years group, the BHPET had the lowest RMSPE (11.83%) and highest accuracy (PW10 = 62.84%, PW20 = 90.8%) (Table 2).

**Table 2:** Performance data for the Broselow tape, PAWPER XL tape, and BHPET.

	Broselow tape	PAWPER XL	BHPET
All patients (n = 792)			
MPE, %	–0.41	–1.4	1.4
MAPE, %	12.47	10.5	10.23
RMSPE, %	17.19	14.43	14.14
LOA (kg)	–11.1 to 9.1	–7.11 to 5.55	–5.41 to 5.66
PW10, %	51.13	59.59	59.6
PW20, %	79.92	85.47	87.12
1–12 months (n = 82)			
MPE, %	–5.21	–1.03	3.04
MAPE, %	14.96	14.39	13.94
RMSPE, %	19	18.2	18.14
LOA (kg)	–3.94 to 2.91	–3.46 to 3.13	–3.06 to 3.33
PW10, %	48.78	54.88	52.44
PW20, %	75.61	74.39	79.27
2–5 years (n = 449)			
MPE, %	3.29	0.36	0.39
MAPE, %	10.95	10.09	10.12
RMSPE, %	15.81	14.36	14.28
LOA (kg)	–5.14 to 5.53	–4.61 to 4.25	–4.41 to 4.11
PW10, %	58.13	63.47	62.36
PW20, %	87.52	89.09	89.76
6–12 years (n = 261)			
MPE, %	–5.23	–4.54	2.62
MAPE, %	14.29	9.98	9.26
RMSPE, %	18.3	12.64	11.83
LOA (kg)	–18.34 to 11.91	–10.73 to 6.72	–7.02 to 8.16
PW10, %	45.59	60.15	62.84
PW20, %	74.33	88.51	90.8

MPE, mean percentage error; MAPE, mean absolute percentage error; RMSPE, root mean square percentage error; LOA, limits of agreement (95% confidence intervals); PW10, percentage of weight estimations within 10% of the actual weight; PW20, percentage of weight estimations within 20% of the actual weight.

Measures of bias, precision, and accuracy are shown by age group.

### Correlation between the actual and estimated weight

**Fig 3:**
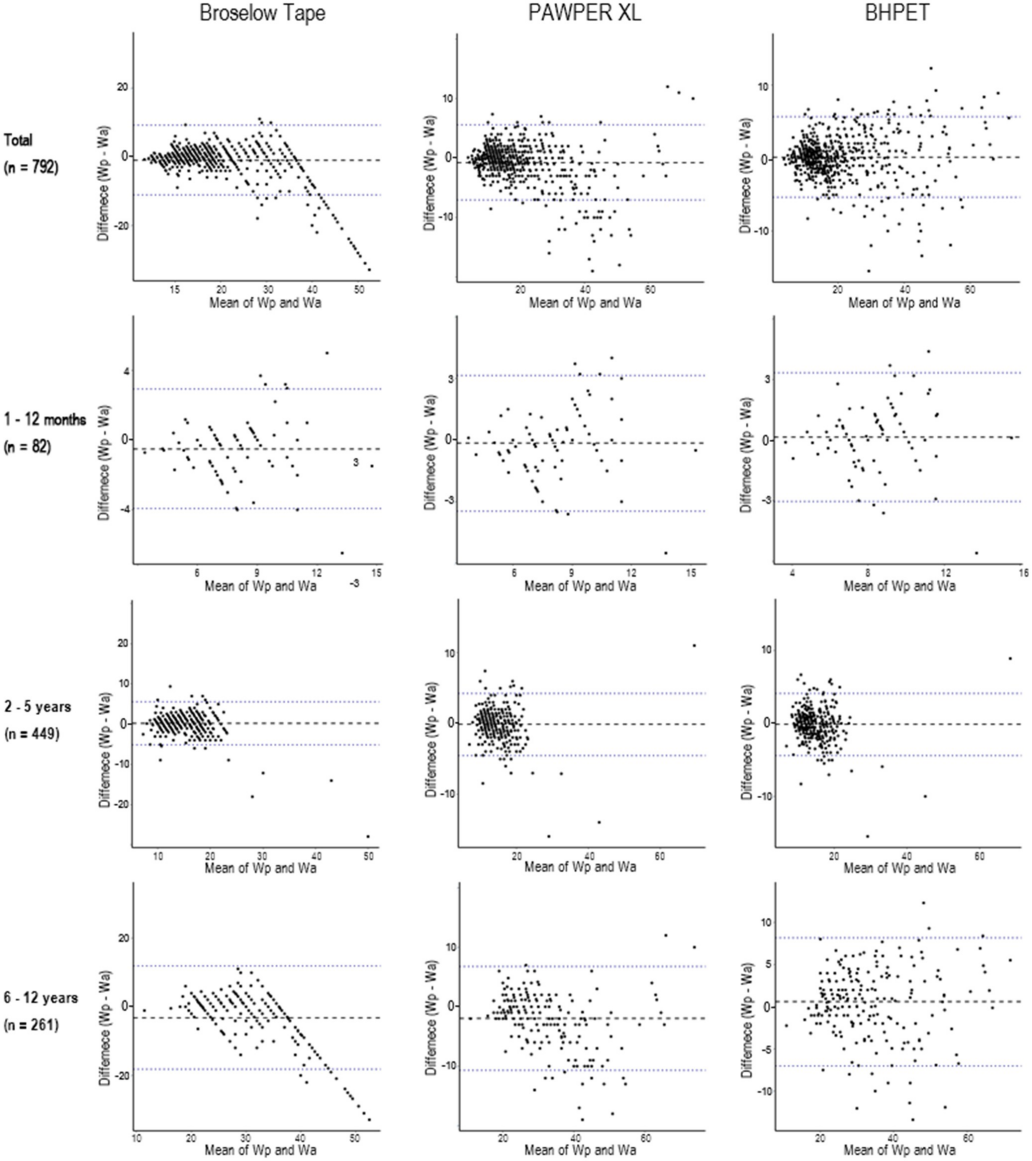
Bland–Altman plot with 95% limits of agreement. Wp, predicted weight; Wa, actual weight.

For all participants, the ICC of the BT, PAWPER XL, and BHPET was 0.931, 0.977, and 0.985, respectively. When calculating weights for each subgroup according to age, the ICC was highest for the BHPET at 1–12 months (0.852), followed by the PAWPER XL (0.85) and BT (0.828). At 2–5 years, the ICC was 0.943 for the BHPET, 0.937 for the PAWPER XL, and 0.887 for the BT. In the 6–12 years group, the ICC was 0.97 for the BHPET, 0.954 for the PAWPER XL, and 0.773 for the BT (P<0.001) (Table 3). According to a Bland–Altman plot, BHPET had a smaller overall mean difference and narrower limits of aggregation compared to the other tapes (Fig 3).

**Table 3:** Correlation between actual and estimated weight by age group.

	BT			PAWPER XL			BHPET		
	ICC	95% CI	*P*-value	ICC	95% CI	*P*-value	ICC	95% CI	*P*-value
All patients (n = 792)	0.931	0.921–0.939	< 0.001	0.977	0.974–0.981	< 0.001	0.985	0.983–0.987	< 0.001
1–12 months (n = 82)	0.828	0.733–0.889	< 0.001	0.85	0.768–0.903	< 0.001	0.852	0.77–0.904	< 0.001
2–5 years (n = 449)	0.887	0.865–0.906	< 0.001	0.937	0.924–0.947	< 0.001	0.943	0.931–0.952	< 0.001
6–12 years (n = 261)	0.773	0.71–0.822	< 0.001	0.954	0.941–0.964	< 0.001	0.97	0.961–0.976	< 0.001

ICC; intraclass correlation coefficient, CI; confidence interval. *P* < 0.05 was taken to indicate statistical significance.

## Discussion

This cross-sectional study developed a weight-estimating tape using the 2017 KNGC as reference values. The performance and accuracy of the BHPET was similar or slightly superior compared to the other tapes. The body weights estimated with the newly developed BHPET for a local pediatric population who visited the emergency department were sufficiently correlated with the actual body weights.

Since pediatric patients visiting the emergency room may require immediate treatment or intervention, accurate weight measurement using an electronic scale is sometimes difficult. However, since most pediatric drug dosages are based on body weight, accurate weight estimation is very important in emergencies [8,10]. If the body weight is under-estimated, there is a risk of under-resuscitation in pediatric patients during cardiac arrest or critical illness due to inadequate drug administration [11,16]. Therefore, the availability of a tool that can predict weight accurately in emergencies is important.

The BHPET was superior in terms of bias, precision, and accuracy than the BT, which has been used as a pediatric weight-estimation tape in emergency situations worldwide. Two-dimensional methods that considered body habitus performed better than the one-dimensional BT method, the estimates of which are based only on length [22,27,30]. When performance was assessed by age subgroup, the BHPET performed better than the BT in all subgroups, especially the 6–12 years group. This was considered to result from the difference in HS according to age group. In the 1–12 months and 2–5 years groups, an HS of 3, which corresponds to the standard weight, was seen in 58.54% and 73.27% of the patients, respectively, compared to 56.32% in the 6–12 years group. There were 33 HS 5–6 patients (12.65%) in the 6–12 years group, representing a higher rate than in the other groups. A previous study reported that the rate of obesity increases with age in schoolchildren, but not in preschoolers [31]. Accordingly, the BHPET, which reflects body habitus, performed better in the school age children (6–12 years) in this study.

Overall, the MAPE and RMSPE values, which reflect overall precision, were slightly better for the BHPET than PAWPER XL. The PW10 should be 60–70%, and the PW20 90–95%, for measurements to be considered accurate [32]. Here, the PAWPER XL and BHPET had PW10 values of 59.59% and 59.6%, and PW20 values of 85.47% and 87.12%, respectively. These results are relatively similar to the accuracy reference values, and the BHPET was slightly better than the PAWPER XL. The ICC between the predicted and actual weight was ≥ 0.9 for all tapes, indicating excellent agreement [29]. However, the ICC for the BT decreased in the older group, while the PAWPER XL and BHPET showed excellent ICC in that group. This difference arose because the PAWPER XL uses the WHO growth chart for reference weights, while the BHPET uses the KCDC growth chart. The WHO growth chart is based on surveys performed in six countries: Brazil, Ghana, India, Norway, Oman, and the USA. Therefore, the application of the WHO chart to other countries is controversial. Some studies used local growth charts instead, which in some cases were partially applied to specific age groups [21]. According to a study in which the WHO growth chart was applied, the WHO growth chart could be used successfully in Korean children up to the age of 24 months, but it was recommended to use the Korean chart for older children [26].

The strength of our study is that the BHPET we developed was made based on the 2017 KNGC, which is the actual growth chart of the Korean pediatric population. The BHPET could help to predict the weight of Korean pediatric patients more accurately than the previously developed BT or PAWPER XL tapes. We expect more precise dosing of drugs could be possible for pediatric patients with this tape.

This study had some limitations. First, it was conducted at a single tertiary hospital in a Korean city. Therefore, its generalizability to populations in other regions may be limited; further analysis with different populations is thus needed Second, since the BHPET was developed using the 2017 KNGC, which is a growth chart used for Korean pediatric population, caution is needed when applying in other countries. Third, most participants clustered around an HS of 3, which might have biased the results due to the uneven distribution of over- and underweight. Thus, more under- and overweight subjects should be analyzed in future studies.

## Conclusion

This prospective cross-sectional study found that the BHPET was as accurate, or slightly superior, to other tapes for estimating the body weight of pediatric participants.
